# Machine learning-based prediction model for postoperative delirium in non-cardiac surgery

**DOI:** 10.1186/s12888-023-04768-y

**Published:** 2023-05-04

**Authors:** Dong Yun Lee, Ah Ran Oh, Jungchan Park, Seung-Hwa Lee, Byungjin Choi, Kwangmo Yang, Ha Yeon Kim, Rae Woong Park

**Affiliations:** 1 Department of Biomedical Informatics, Ajou University School of Medicine, 206, World cup-ro, Yeongtong-gu, Suwon, Korea; 2 Department of Medical Sciences, Graduate School of Ajou University, Suwon, Republic of Korea; 3 Department of Anesthesiology and Pain Medicine, Samsung Medical Center, Sungkyunkwan University School of Medicine, 81 Irwon-ro, Gangnam-gu, Seoul, Korea; 4 Department of Anesthesiology and Pain Medicine, Kangwon National University Hospital, Chuncheon, Korea; 5 Rehabilitation & Prevention Center, Heart Vascular Stroke Institute, Samsung Medical Center, Sungkyunkwan University School of Medicine, Seoul, Korea; 6 Department of Biomedical Engineering, Seoul National University College of Medicine, Seoul, Korea; 7 Center for Health Promotion, Samsung Medical Center, Sungkyunkwan University School of Medicine, Seoul, Korea; 8 Department of Anesthesiology and Pain Medicine, Ajou University School of Medicine, Suwon, Korea

**Keywords:** Machine learning, Delirium, Non-cardiac surgery

## Abstract

**Background:**

Postoperative delirium is a common complication that is distressing. This study aimed to demonstrate a prediction model for delirium.

**Methods:**

Among 203,374undergoing non-cardiac surgery between January 2011 and June 2019 at Samsung Medical Center, 2,865 (1.4%) were diagnosed with postoperative delirium. After comparing performances of machine learning algorithms, we chose variables for a prediction model based on an extreme gradient boosting algorithm. Using the top five variables, we generated a prediction model for delirium and conducted an external validation. The Kaplan–Meier and Cox survival analyses were used to analyse the difference of delirium occurrence in patients classified as a prediction model.

**Results:**

The top five variables selected for the postoperative delirium prediction model were age, operation duration, physical status classification, male sex, and surgical risk. An optimal probability threshold in this model was estimated to be 0.02. The area under the receiver operating characteristic (AUROC) curve was 0.870 with a 95% confidence interval of 0.855–0.885, and the sensitivity and specificity of the model were 0.76 and 0.84, respectively. In an external validation, the AUROC was 0.867 (0.845–0.877). In the survival analysis, delirium occurred more frequently in the group of patients predicted as delirium using an internal validation dataset (*p* < 0.001).

**Conclusion:**

Based on machine learning techniques, we analyzed a prediction model of delirium in patients who underwent non-cardiac surgery. Screening for delirium based on the prediction model could improve postoperative care. The working model is provided online and is available for further verification among other populations.

**Trial registration:**

KCT 0006363.

**Supplementary Information:**

The online version contains supplementary material available at 10.1186/s12888-023-04768-y.

## Background

Delirium is characterized by acute confusion that is commonly reversible and preventable [1]. Delirium is stressful for patients, families, and healthcare providers and leads to increased duration of hospital stay, healthcare costs, complications, readmission rate, and in-hospital mortality [2–4]. In surgical patients, delirium is a common complication, with a prevalence varying widely from 5 to 40% based on surgery type [5]. Previous studies reported that screening of delirium can lead to increased rate of diagnosis and early intervention that reduce duration and complications of delirium [6]. Effectiveness of appropriate perioperative interventions based on delirium prediction have been described [7]. Although various methods have been introduced, prediction of postoperative delirium remains challenging, and a widely accepted prediction tool does not yet exist [8]. Numerous factors other than surgery type that reflect cerebral vulnerability and exogenous neurocognitive stressors are involved in the occurrence of delirium. Furthermore, an overlap exists between predisposing and precipitating factors of postoperative delirium, complicating prevention of delirium during postoperative care.

To overcome this issue, we considered a machine learning technique that has recently gained attention in studies evaluating predictors. The machine learning technique can handle numerous variables in nonlinear and highly interactive ways [9]. In a recent study, the machine learning model outperformed traditional clinician-based regression models in predicting postoperative delirium [10]. However, this model was not externally validated in other populations. Another previous model was based on a relatively small number of patients and limited to an older age group [11]. Therefore, our study used a larger amount of real-world data of consecutive adult patients who underwent surgery in a large tertiary center between January 2011 and June 2019 to generate a prediction model. Furthermore, the model was validated using a dataset from another institution and is provided online for further verification.

## Methods

### Ethics

This study was conducted in accordance with the Declaration of Helsinki and was reported following the Strengthening the Reporting of Observational Studies in Epidemiology. Because the registry is curated in a de-identified form, the Institutional Review Board of Samsung Medical waived approval (Samsung Medical Center, 81 Irwon-ro, Gangnam-gu, Seoul, Korea, 2021-06-078 Chairperson Prof. SW Park) on 26th June 2021, and written informed consent from participants was also waived. Use of the dataset for external validation was approved by the Institutional Review Board of Ajou University Hospital (World cup-ro, Yeongtong-gu, Suwon, Korea, AJIRB-MED-MDB-21-662 Chairperson Prof. SU Han).

### Data curation and study population

This study used Samsung Medical Center-Non-Cardiac operation (SMC-NoCop) registry (cris.nih.go.kr; registration number KCT 0006363; registration day 21/07/2021). The registry is a single-center de-identified cohort of 203,787 consecutive patients 18 years of age and older who underwent non-cardiac surgery at Samsung Medical Center, Seoul, Korea, between January 2011 and June 2019. The registry is based on raw data extracted by the Clinical Data Warehouse Darwin-C, an electronic system that enables investigators to search and retrieve de-identified medical records of the institutional electronic archive system. This system contains electronic hospital records of more than 4 million patients and comprises more than 900 million laboratory findings and 200 million prescriptions. For deaths outside the institution, the system uses data from the National Population Registry of the Korea National Statistical Office.

Data from Ajou University Medical Center were used for external validation. We curated data between January 2011 and October 2021, using the same recruitment criteria and included 101,582 patients in the external validation dataset.

### Predictors

A total of 54 predictor variables obtained from a preoperative evaluation sheet was provided as input to each model (Additional file 1: Table S1). Investigators independent from this study organized relevant preoperative variables including demographic data, underlying diseases, and information from blood laboratory tests. In addition, we used International Classification of Diseases-10 codes to organize preoperative diagnosis and estimated Charlson Comorbidity Index [12]. The risks of surgical procedures were stratified according to the European Society of Cardiology (ESC)/European Society of Anaesthesiology (ESA) guidelines on non-cardiac surgery [13]. The American Society of Anesthesiologists (ASA) Physical Status Classification was classified by attending anesthesiologists and extracted from the preoperative evaluation sheet [14].

### Study endpoints and definitions

The primary endpoint was postoperative delirium diagnosed by a psychiatrist using Diagnostic Statistical Manual (DSM) criteria during the first 30 postoperative days. Patients assessed for acute confusion or behavioral change using the confusion assessment method (CAM) were referred to the department of psychiatry at the discretion of attending clinicians. Specifically, CAM is based on the four features of delirium including acute onset and fluctuating course, inattention, disorganised thinking, and altered level of consciousness. CAM considers patients delirious when acute onset, fluctuating course, and inattention are accompanied by either disorganized thinking or altered level of consciousness. For a referred patient, the attending psychiatrist uses Diagnostic Statistical Manual (DSM) criteria to assess the patient for delirium. To ensure a first-time diagnosis of delirium, we excluded patients who had history of delirium or dementia preoperatively.

### Model development

We compared the performance of prediction models created by four machine learning algorithms: extreme gradient boosting (XGB), random forest (RF), logistic regression (LR), and Naive Bayes (NB). Further details of machine learning algorithms are presented in Additional file 1: Table S2.

### Model evaluation

We calculated four metrics to evaluate predictive models: accuracy, F1 score, area under the precision and recall curve (AUPRC), and area under the receiver operating characteristic curve (AUROC). We optimized the hyper-parameters based on a grid search using the AUROC curve and the five-fold cross-validation used during model development. We divided the data into training and test models using a stratified random split with a constant probability of an event. Postoperative delirium was an event in this study, in which 80% of the data were reserved for creating the machine learning model and the remaining 20% for the testing model. In addition, we included calibration metrics of calibration plot, calibration slope, intercept, Spiegelhalter z statistic, and Brier score. With the Spiegelhalter z statistic, *P* > 0.05 indicates a well calibrated model [15]. We used the maximal Youden index to select the optimal cut-off value in each prediction model and calculated the corresponding accuracy [16]. We also generated a case balanced dataset for an internal validation.

The SHapley Additive exPlanations (SHAP) summary plot was used to present feature importance. The effect of each feature on postoperative delirium was presented as a SHAP value representing the importance of a variable by deriving a marginal distribution and weighted average with all but the variable of interest fixed [17]. The Shapley value is defined as the average marginal contribution of a feature value across all possible feature coalitions. Under this definition, a Shapley value for a given feature value can be interpreted as the difference between the actual prediction and the average prediction for the entire data set. The SHAP summary plot sorts features in descending order based on effects on postoperative delirium. One dot on each variable line represents one patient, and the horizontal location indicates the level of association between the feature and outcome. The right side is where the SHAP value is > 0, and variable-specific SHAP values > 0 indicate increased risk of outcome.

A sub-analysis using an internal validation dataset was conducted to validate the predicted delirium outcomes. Among the sub-analysis patients, patients were divided into high-risk and low-risk patient groups according to the finalized prediction model. The Kaplan–Meier and Cox survival analyses were used to analyse the difference of delirium occurrence in the high-risk patient group versus low-risk patient group.

### External validation

To confirm the validity of the model performance, we conducted external validation using a different dataset from Ajou University Medical Center. The best performance model using the selected five variables was validated.

### Statistical analysis

The differences between patients with and without postoperative delirium were determined. Continuous features are presented as mean ± standard deviation or median with interquartile range, and comparisons were conducted using *t*-test or Mann-Whitney test, as applicable. Categorical features are presented as number and percentage, and differences were evaluated using chi-square or Fisher’s exact test. Survival analysis was performed using the survival package, and P values for comparing the survival rates were obtained using the log-rank test. Analysis was performed using R 4.1.0 (Vienna, Austria; http://www.R-project.org/).

## Results

### Baseline characteristics

We excluded 413 patients who were diagnosed with delirium or dementia preoperatively. A total of 203,374 patients was included for model development, and postoperative delirium was diagnosed in 2,865 (1.4%) patients. The baseline characteristics of patients with and without postoperative delirium are presented in Table 1. Patients with delirium were predominantly male, older, had higher ASA Physical Status Classification, and tended to show a higher incidence of psychologic disorder, underlying disease, and electrolyte imbalance. Intraoperatively, patients with delirium more frequently underwent emergency surgery under general anesthesia with longer operation duration (Table 2). Mortality during the first year after surgery was higher in patients with delirium (2.7% vs. 17.0%).

**Table 1:** Baseline characteristics of patients with and without postoperative delirium

	No delirium (*N* = 200,509)	Delirium (*N* = 2,865)	*p* value
Male	85978 (42.9)	1916 (66.9)	< 0.001
Age	52.6 (± 15.2)	65.0 (± 14.4)	< 0.001
Body mass index	24.2 (± 3.6)	23.6 (± 3.7)	< 0.001
ASA physical status			< 0.001
I	87777 (43.8)	259 (9.0)	
II	101562 (50.7)	1580 (55.1)	
III	10500 (5.2)	900 (31.4)	
IV	529 (0.3)	122 (4.3)	
V	141 (0.1)	4 (0.1)	
Psychiatric disorder, any	6609 (3.3)	348 (12.1)	< 0.001
Mood disorder	2779 (1.4)	157 (5.5)	< 0.001
Schizophrenia	189 (0.1)	40 (1.4)	< 0.001
Alcoholic use disorder	129 (0.1)	15 (0.5)	< 0.001
Substance abuse (without alcohol)	44 (0.0)	3 (0.1)	0.02
Sleep disorder	2779 (1.4)	83 (2.9)	< 0.001
Personality disorder	59 (0.0)	3 (0.1)	0.08
Current alcohol	40107 (20.0)	421 (14.7)	< 0.001
Current smoking	15248 (7.6)	310 (10.8)	< 0.001
Previous disease			
Hypertension	50346 (25.1)	1248 (43.6)	< 0.001
Diabetes	22810 (11.4)	722 (25.2)	< 0.001
Chronic kidney disease	3210 (1.6)	182 (6.4)	< 0.001
Dialysis	869 (0.4)	97 (3.4)	< 0.001
Charlson comorbidity index	0.2 (± 0.6)	0.5 (± 1.1)	< 0.001
Stroke	4022 (2.0)	239 (8.3)	< 0.001
Coronary artery disease	3970 (2.0)	165 (5.8)	< 0.001
Coronary revascularization			
Percutaneous intervention	2939 (1.5)	133 (4.6)	< 0.001
Bypass graft	399 (0.2)	25 (0.9)	< 0.001
Heart failure	579 (0.3)	44 (1.5)	< 0.001
Arrhythmia	2832 (1.4)	154 (5.4)	< 0.001
Atrial fibrillation	1776 (0.9)	129 (4.5)	< 0.001
Peripheral artery disease	533 (0.3)	43 (1.5)	< 0.001
Aortic disease	629 (0.3)	67 (2.3)	< 0.001
Valvular heart disease	305 (0.2)	12 (0.4)	< 0.001
Chronic obstructive pulmonary disease	3465 (1.7)	158 (5.5)	< 0.001
Preoperative blood laboratory tests			
Hemoglobin, g/dl	13.3 (± 1.8)	12.2 (± 2.2)	< 0.001
Creatinine, mg/dL	0.9 (± 0.8)	1.3 (± 1.7)	< 0.001
Preoperative electrolytes			
Hypernatremia	1395 (0.7)	34 (1.2)	< 0.001
Hyponatremia	6199 (3.1)	442 (15.4)	< 0.001
Hyperkalemia	1247 (0.6)	67 (2.3)	< 0.001
Hypokalemia	2488 (1.2)	153 (5.3)	< 0.001
Hyperphosphatemia	5576 (2.8)	153 (5.3)	< 0.001
Hypophosphatemia	4477 (2.2)	205 (7.2)	< 0.001
Hyperchloremia	21110 (10.5)	667 (23.3)	< 0.001
Hypochloremia	3593 (1.8)	238 (8.3)	< 0.001

Data are presented as n (%) or mean (± standard deviation)

*ASA* American Society of Anesthesiologists

**Table 2:** Operative variables of patients with and without postoperative delirium

	No delirium (*N* = 200,509)	Delirium (*N* = 2,865)	*p* value
General anesthesia	173,540 (86.5)	2599 (90.7)	< 0.001
Emergency operation	13,765 (6.9)	648 (22.6)	< 0.001
Operation duration, min	130.4 (± 98.6)	233.3 (± 174.5)	< 0.001
Surgical risk			< 0.001
Mild	78,787 (39.3)	414 (14.5)	
Intermediate	110,034 (54.9)	1696 (59.2)	
High	11,688 (5.8)	755 (26.4)	
Surgery types			
Neuroendocrine	13,050 (6.5)	21 (0.7)	< 0.001
Lung	11,743 (5.9)	324 (11.3)	< 0.001
Head & Neck	30,491 (15.2)	514 (17.9)	< 0.001
Breast	17,629 (8.8)	37 (1.3)	< 0.001
Stomach	12,492 (6.2)	99 (3.5)	< 0.001
Hepatobiliary	16,697 (8.3)	499 (17.4)	0.97
Colorectal	13,635 (6.8)	283 (9.9)	0.32
Urology	18,431 (9.2)	159 (5.5)	< 0.001
Gynecology	24,487 (12.2)	43 (1.5)	< 0.001
Bone & Skin etc.	41,854 (20.9)	886 (30.9)	< 0.001

Data are presented as n (%) or mean (± standard deviation)

Surgical risk was stratified according to 2014 European Society of Cardiology/European Society of Anaesthesiology guidelines

### Development of prediction model

**Fig. 1:**
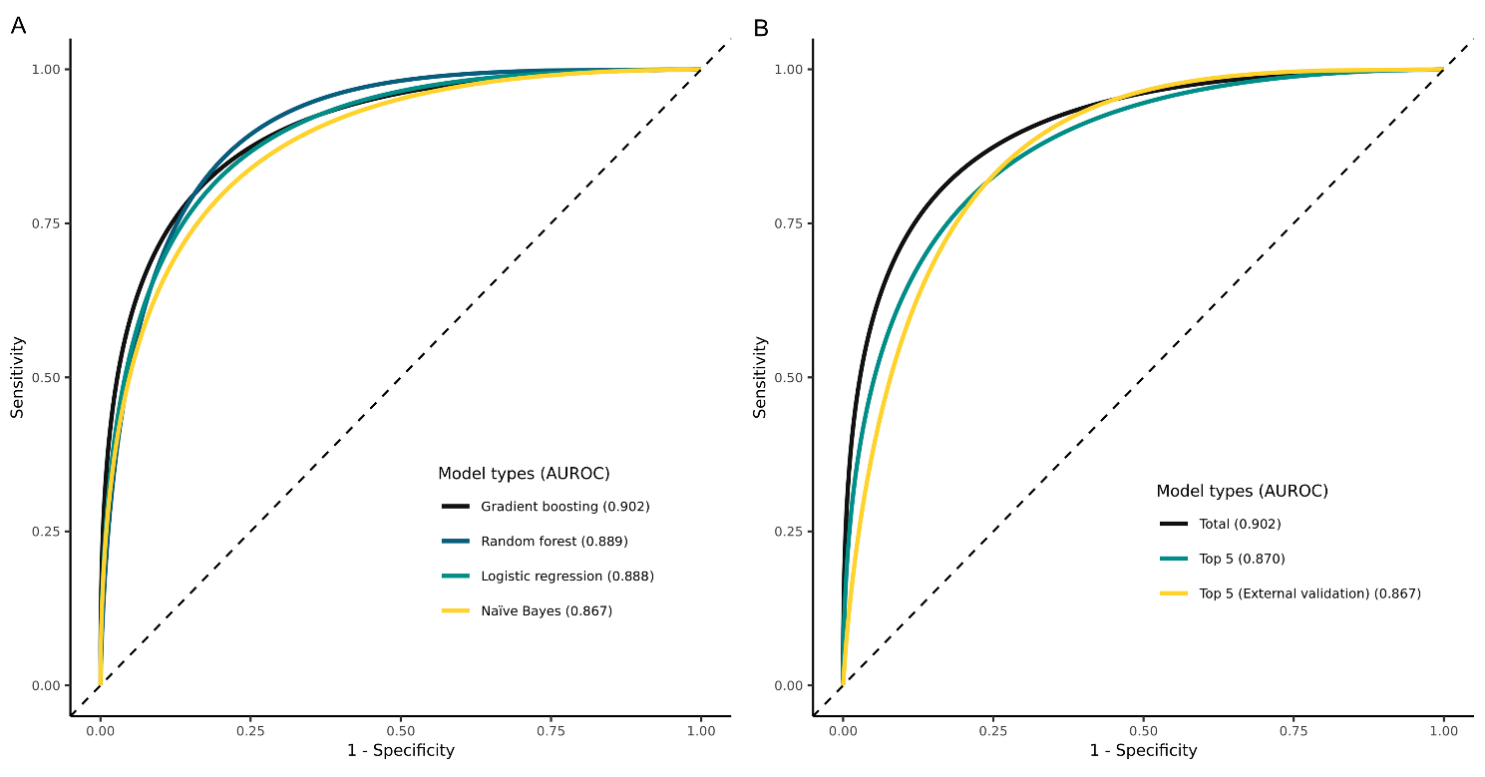
Receiver operating characteristic (ROC) curves of the prediction model: A, ROC curves for postoperative delirium according to different machine learning algorithm using an internal validation dataset, B, ROC curves for postoperative delirium of the extreme gradient boosting (XGB) algorithm according to number of retained variables using internal validation dataset and external validation dataset

The AUROCs for the XGB, RF, LR, and NB algorithms were 0.902 (0.889–0.913), 0.889 (0.813–0.949), 0.888 (0.870–0.898), and 0.867 (0.845–0.877), respectively (Fig. 1). In terms of the performance metrics of accuracy, AUPRC, AUROC, and F1 score, the XGB model and RF model showed comparable performances (XGB: 0.855, 0.170, 0.902, 0.136; RF: 0.974, 0.080, 0.889, 0.186; LR: 0.828, 0.149, 0.888, 0.127; NB: 0.828, 0.105, 0.867, 0.122, respectively). We chose XGB because even though the F1 score of RF was higher than XGB, the AUPRC of RF was much lower than XGB. The performance metrics of each model are summarized in Additional file 1: Table S3. We selected the XGB algorithm for the final model.

### Development of prediction model using selected variables

The XGB algorithm is a decision tree-based ensemble model using a gradient boosting framework and the Shapley value framework known to fairly evaluate performance [17].

**Fig. 2:**
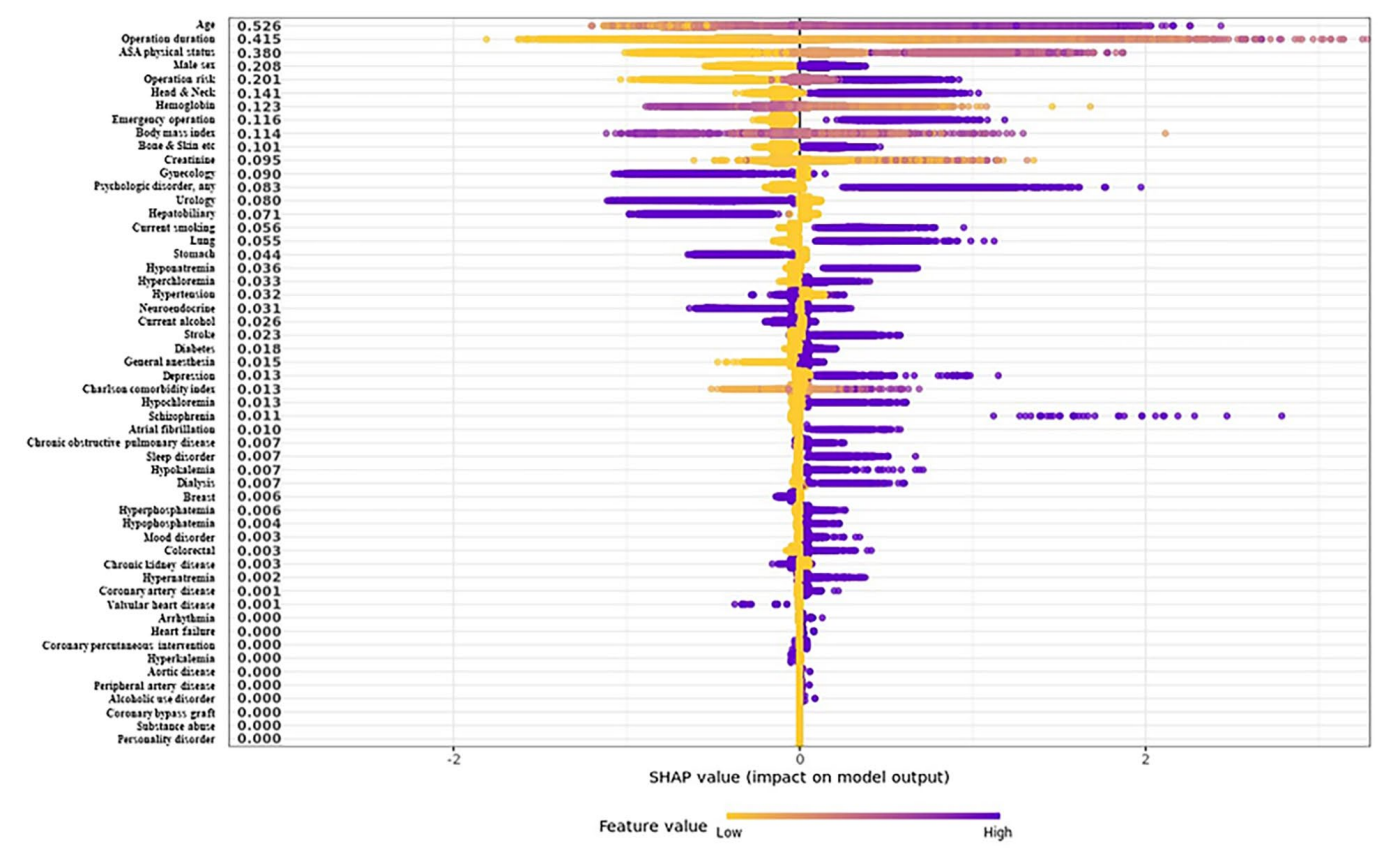
SHapley Additive exPlanations (SHAP) summary plot representing the results of a machine learning-based extreme gradient boosting (XGB) algorithm

The SHAP summary plot was generated based on the results of the XGB model (Fig. 2). For practical use of prediction models in clinical practice, we eliminated variables based on the degree of effect on the outcome and selected the top five variables with a SHAP value > 0.2 for the final model. The top five variables with SHAP value > 0.2 were age (0.526), operation duration (0.415), ASA Physical Status Classification (0.380), male sex (0.208), and surgical risk according to ESC/ESA guidelines (0.201). We generated a prediction model for delirium based on these variables. The AUROC of the prediction model using the selected variables was 0.870 (0.855–0.885; Fig. 1). Other performance metrics of the prediction model were accuracy of 0.834, AUPRC of 0.148, and F1 score of 0.114 (Additional file 1: Table S3). In the internal validation using a case balanced dataset, AUPRC, AUROC, and F1 score were improved (0.855, 0.860, and 0.807, respectively).

We used leveraging Shiny, an application-building package from R, to allow others to freely access the application *via* a public link. The optimal probability threshold based on maximum Youden index was estimated to be 0.020 in this model. Applying this threshold, the sensitivity and specificity of the model were 0.76 and 0.84, respectively. A functioning version of the model is provided online athttps://psyshiny.shinyapps.io/shiny/. When values for each of the top five variables for target patients are entered, the probability for delirium is shown as an output.

### External validation of prediction model

The external validation dataset represented 101,582 patients. Postoperative delirium developed in 327 (0.3%) patients. The model achieved an AUROC of 0.867 (0.845–0.877) in the external validation dataset (Fig. 1). Other external validation performance metrics of the prediction model using the selected variables were accuracy of 0.745, AUPRC of 0.062, and F1 score of 0.064 (Additional file 1: Table S2).

### Kaplan-Meier analysis of stratified patients

**Fig. 3:**
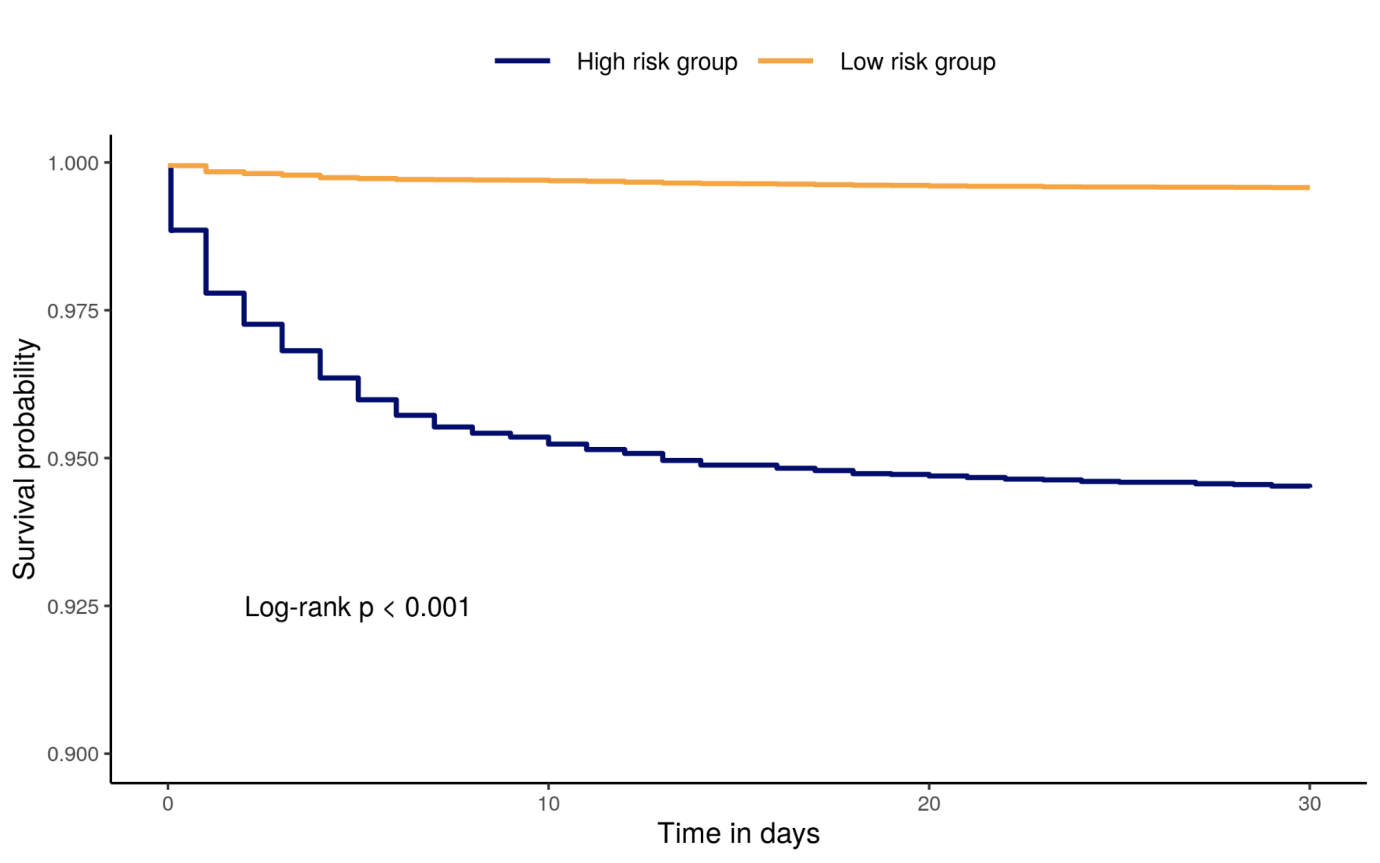
Kaplan-Meier survival analysis of stratified patients using an internal validation dataset

Figure 3 shows the clinical benefit of using the prediction model for improving early detection by supporting delirium screening. Survival analysis showed that delirium occurred more frequently in high-risk patient group than in low-risk patient group (log-rank, *p* < 0.001). The hazard ratio was 13.3 (95% CI 10.99–16.13, *p* < 0.001).

### Calibration of prediction model

We showed that both the XGB model using all variables and the XGB model using selected variables were well calibrated (Total: Spiegelhalter z = − 0.05; *p* = 0.051; Top five: Spiegelhalter z = − 0.27; *p* = 0.064, respectively). The calibration plot of each model is shown in Additional file 1: Fig. S1.

## Discussion

In this study, we demonstrated a prediction model for delirium after non-cardiac surgery. We selected the five variables age, operation duration, ASA Physical Status Classification, sex, and operation risk based on machine learning techniques. The incidence of postoperative delirium was 1.4% in the data set, and the applied models showed fair performance for delirium prediction. Our final prediction model achieved an AUROC value of 0.870 (0.855–0.885). AUPRC and F1 score was relatively low owing to case imbalanced nature of the dataset, but these metrics were improved in an internal validation with case balanced dataset. This model was validated and showed similar predictive power in a separate cohort. The clinical benefit of the prediction model for screening postoperative delirium was also evaluated.

Delirium is an acute state of confusion accompanied by fluctuating awareness, disorientation, memory impairment, disturbances of perception, and disorganized thinking [5]. Although these symptoms are mostly reversible, the condition is distressing and can lead to serious and costly consequences such as increased duration of hospital stay and higher rates of re-admission, complication, and mortality during hospital stay [2–4]. Our data also showed that one-year mortality was significantly increased in patients diagnosed with delirium. Postoperative delirium has been reported to be preventable in nearly 40% of patients [1, 18], and identifying individuals at higher risk is the first step to modify precipitating factors and allow interventions to be appropriately targeted. This study applied relatively strict criteria for delirium as diagnosed by psychiatrists to distinguish symptoms from those of remnant anesthetics immediately after operation. From a clinical perspective, another strength of our prediction model is it can be widely applied over a broad spectrum of non-cardiac surgical procedures with a small number of readily available variables. Furthermore, we conducted an external validation and showed similar prediction capability in a dataset with significantly lower prevalence of delirium and an internal validation with balanced dataset. The difference in delirium incidence between institutions has been reported in Korea [19], and validation in a dataset with different features is desirable when evaluating the generalizability of the model [20].

Causes of delirium include any pathophysiological stressor that affects cerebral functioning [2]. Multicomponent risk factors that reflect an interplay between cerebral vulnerability and neurocognitive stressors during pre- and intraoperative periods should be considered for prediction of postoperative delirium. Several scoring systems based on traditional regression models have been proposed to predict postoperative delirium [21, 22]. However, these models showed inadequate and variable capabilities, and a universally accepted system does not exist in daily practice [8]. In this study, we adopted machine learning techniques that can handle a complex relationships of numerous variables with nonlinear interactions to demonstrate a prediction model for postoperative delirium [23]. Furthermore, we compared several algorithms of machine learning techniques and chose XGB for its highest performance [24, 25].

In the field of medicine, artificial intelligence results, such as those of machine learning techniques, should be interpreted based on clinical suitability. We selected variables according to SHAP feature importance based on Shapley value, which is computationally fast and has good theoretical properties [17]. The variables retained in our model were previously associated with delirium. Age is the most well-known risk factor for delirium in any clinical situation [2, 26]. In a number of previous studies, male sex was reported a risk factor for postoperative delirium [8, 27, 28]. Delirium is caused by deterioration of homeostasis and physical status, and these conditions are more likely to occur during higher-risk surgery with longer duration [29, 30]. The ASA Physical Status Classification has long been widely used to stratify the risk of patients undergoing surgical procedures [14]. Finally, the surgical risk in our model was stratified according to the ESC/ESA guidelines on non-cardiac surgery [13], which group types of surgery based on postoperative mortality and are well known to reflect the metabolic burden of surgical procedures.Our model is clinically explainable, has only five variables, and shows high performance. The delirium prediction risk is easily calculated from the URL (https://psyshiny.shinyapps.io/shiny/). Specifically, the risk of delirium increased with age and operation duration. ASA Physical Status Classification score also increased the risk of delirium as the score increases, e.g. severe systemic disease with a score of 3 is more risky than normal healthy patients with a score of 1. The risk was also higher for men than women and for high-risk surgeries. Surgical risk was categorized according to the ESC/ESA guidelines: low-risk surgeries with a score of 0 included procedures such as debridement, simple sutures, and mastectomy, while high-risk surgeries with a score of 2 included aortic valve replacement, perforated appendectomy, and amputation. In addition, the patients in high-risk group were more likely to develop postoperative delirium than those in low-risk group. The model can be helpful for effectively screening and preventing postoperative delirium as well as to determine further treatment by psychiatric or rehabilitation professionals with limited resources [10]. Furthermore, the model showed modest calibration to reflect reality despite case imbalance. Previous models that originated from a case-imbalanced dataset showed a limitation due to poor calibration [31]. Further studies for clinical applications are needed to identify the potential feasibility of this model.

There are several limitations that need to be considered when interpreting our results. First, the variables of the models are clinically relevant, but causality cannot be confirmed due to the nature of the retrospective data. Particularly. the wide standard deviation in operation duration was observed in both groups, which may be a reflection of the real-world variability that occurs in surgical practice and could impact the interpretation of our findings. Second, institutional protocol for perioperative care can vary between departments and could have changed during the long study period. Despite the institutional protocol, decisions often were made at the discretion of attending clinicians. Third, the results cannot be generalized to other patient groups because ethnic differences were not considered. Fourth, the data were imbalanced with a low incidence of delirium, resulting in low sensitivity of the model. However, this low prevalence was caused by the fact that the diagnosis code was recorded only when the patient was consulted by the psychiatry department. Furthermore, this study included a large number of patients, compared to other studies, and it might lead to low prevalence inevitably. Because there was no systematic screening of patients for delirium postoperatively, it seems that many cases were not noticed in real-world setting. That’s why this prediction model for screening is needed and will need to be verified by other datasets in the future. Fifth, other delirium risk factors from previous studies were not included in this study. For example, the preoperative status of cognitive function, sleep evaluation, emotional status, current medication, etc. were not included as variables. However, the variables used in this study are those that are routinely recorded on preoperative assessment sheets. In order to develop a clinically useful model, model development was performed using routinely recorded information. Also, our study was to predict postoperative delirium with preoperative variables, so we were not able to include perioperative or postoperative variables. Last, factors retained in the model were mostly non-modifiable, and prevention or treatment strategies could not be proposed. Despite these limitations, ours is the first study to identify risk factors for postoperative delirium in non-cardiac surgery using a machine learning algorithm and a proven prediction model.

## Conclusion

We selected five variables using machine learning techniques and demonstrated a prediction model for delirium in patients undergoing non-cardiac surgery. This model could be useful for predicting postoperative delirium and identifying high-risk patients in advance.

## Electronic supplementary material

Below is the link to the electronic supplementary material.

**Supplementary Table S1.** Variables. **Supplementary Table S2.** Description of Machine Learning Algorithms. **Supplementary Table S3.** Performance Metrics of Models. **Supplementary Figure S1.** The Calibration Plot of Models

## Data Availability

The datasets used and/or analysed during the current study available from the corresponding author on reasonable request.

## Abbreviations

ASA: American Society of Anesthesiologists
AUPRC: area under the precision and recall curve
AUROC: area under the receiver operating characteristic curve
CAM: confusion assessment method
ESA: European Society of Anaesthesiology
ESC: European Society of Cardiology
LR: logistic regression
NB: Naive Bayes
RF: random forest
SHAP: SHapley Additive exPlanations
SMC-NoCop: Samsung Medical Center-Non-Cardiac operation
XGB: extreme gradient boosting
