# Supplementary material for: Machine learning-based prediction model for postoperative delirium in non-cardiac surgery
Source: BMC Psychiatry. 2023 May 4;23:317. doi: 10.1186/s12888-023-04768-y (PMC10161528; doi:10.1186/s12888-023-04768-y)
Supplement: Supplementary file 1 — Supplementary Table S1. Variables. Supplementary Table S2. Description of Machine Learning Algorithms. Supplementary Table S3. Performance Metrics of Models. Supplementary Figure S1. The Calibration Plot of Models [file 12888_2023_4768_MOESM1_ESM.docx]

**Supplementary Table S1. Variables**

| **Demographic data** | **Male** |
| --- | --- |
|  | **Age** |
|  | **Body mass index** |
| Physical function and underlying disease | ASA physical status |
|  | I |
|  | II |
|  | III |
|  | IV |
|  | V |
|  | Psychiatric disorder, any |
|  | Mood disorder |
|  | Schizophrenia |
|  | Alcoholic use disorder |
|  | Substance abuse (without alcohol) |
|  | Sleep disorder |
|  | Personality disorder |
|  | Current alcohol |
|  | Current smoking |
|  | Previous disease |
|  | Hypertension |
|  | Diabetes |
|  | Chronic kidney disease |
|  | Dialysis |
|  | Charlson comorbidity index |
|  | Stroke |
|  | Coronary artery disease |
|  | Coronary revascularization |
|  | Percutaneous intervention |
|  | Bypass graft |
|  | Heart failure |
|  | Arrhythmia |
|  | Atrial fibrillation |
|  | Peripheral artery disease |
|  | Aortic disease |
|  | Valvular heart disease |
|  | Chronic obstructive pulmonary disease |
| Preoperative blood laboratory tests and electrolytes | Hemoglobin, g/dl |
|  | Creatinine, mg/dL |
|  | Creatinine, mg/dL |
|  | Hypernatremia |
|  | Hyponatremia |
|  | Hyperkalemia |
|  | Hypokalemia |
|  | Hyperphosphatemia |
|  | Hypophosphatemia |
|  | Hyperchloremia |
|  | Hypochloremia |
| Operative factors | General anesthesia |
|  | Emergency operation |
|  | Operation duration, min |
|  | Surgical risk |
|  | Mild |
|  | Intermediate |
|  | High |
|  | Surgery types |
|  | Neuroendocrine |
|  | Lung |
|  | Head & Neck |
|  | Breast |
|  | Stomach |
|  | Hepatobiliary |
|  | Colorectal |
|  | Urology |
|  | Gynecology |
|  | Bone & Skin etc |

**Supplementary Table S2. Description of Machine Learning Algorithms**

| **Model** | **Description** |
| --- | --- |
| **Gradient boosting** | The eXtreme Gradient Boosting (XGBoost) algorithm is a decision tree-based model on the training dataset. XGBoost starts with a simple initial model and its residuals/misclassifications are iteratively improved in subsequent models searching from among all available predictors to try to minimize misclassification. XGBoost was commonly chosen for its interpretability of results and robustness to overfitting. |
| **Random forest** | The random forest model uses classification tress as building blocks to construct prediction models. A random forest model is developed by only considering a small subset of the predictors each time it splits. This process results in a reduction of the correlation among the trees, thus making the average of the resulting tress less variable and more reliable |
| **Logistic regression** | The logistic regression is one of the most commonly used statistical methods, and previous models using logistic regression showed good performances. Logistic regression allows for multivariate analysis and modelling of a binary dependent variable. The multivariate analysis estimates coefficients for each predictor. |
| **Naive Bayes** | The Naive Bayes model is based on the Bayes’ theorem with the conditional independence assumptions, and this calculates each variable independently. Because the Naive Bayes model can handle noisy data and have low risk of over-fitting, this model has been widely applied for the prediction. |

**Supplementary Table S3. Performance Metrics of Models**

|  | **AUROC** | **AUPRC** | **Accuracy** | **F1 score** |
| --- | --- | --- | --- | --- |
| Extreme gradient boosting | 0.902 | 0.17 | 0.855 | 0.136 |
| Random forests | 0.889 | 0.08 | 0.974 | 0.186 |
| Logistic regression | 0.888 | 0.149 | 0.828 | 0.127 |
| Naive Bayes | 0.867 | 0.105 | 0.828 | 0.122 |
| Top 5 model | 0.87 | 0.148 | 0.834 | 0.114 |
| Top 5 model of external validation | 0.867 | 0.062 | 0.745 | 0.064 |

Abbreviations: AUROC, area under the receiver operating characteristic curve; AUPRC, area under the precision and recall curve.

**Supplementary Figure S1. The Calibration Plot of Models**

**
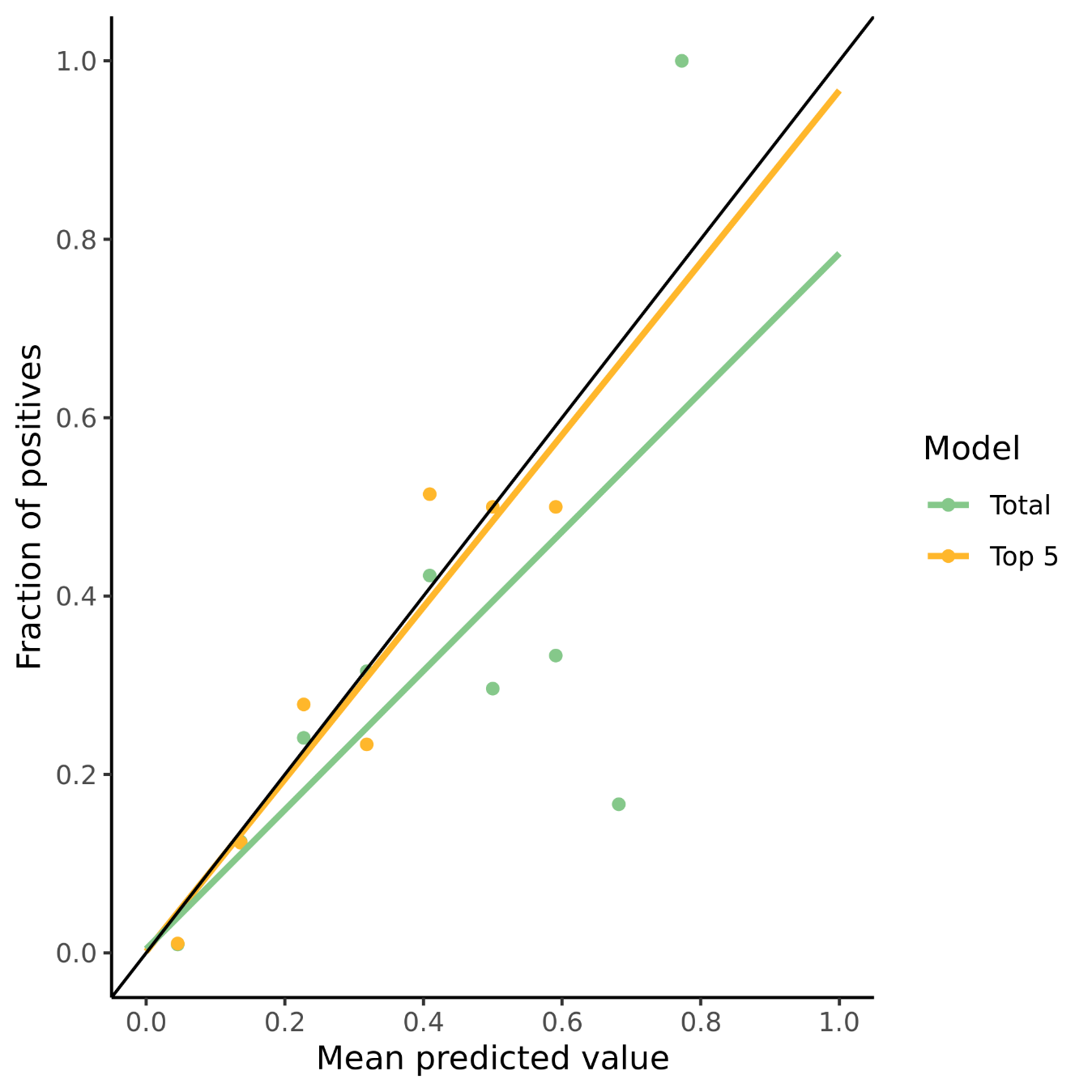
**
